# Isolation and Identification of *Lactobacillus plantarum* HFY05 from Natural Fermented Yak Yogurt and Its Effect on Alcoholic Liver Injury in Mice

**DOI:** 10.3390/microorganisms7110530

**Published:** 2019-11-05

**Authors:** Ruokun Yi, Fang Tan, Wei Liao, Qiang Wang, Jianfei Mu, Xianrong Zhou, Zhennai Yang, Xin Zhao

**Affiliations:** 1 Chongqing Collaborative Innovation Center for Functional Food, Chongqing Engineering Research Center of Functional Food, Chongqing Engineering Laboratory for Research and Development of Functional Food, Chongqing University of Education, Chongqing 400067, China; yirk@cque.edu.cn (R.Y.); mujianfei@foods.ac.cn (J.M.); zhouxr@foods.ac.cn (X.Z.); 2 Beijing Advanced Innovation Center for Food Nutrition and Human Health, Beijing Technology and Business University (BTBU), Beijing 100048, China; 3 Beijing Engineering and Technology Research Center of Food Additives, Beijing Technology and Business University (BTBU), Beijing 100048, China; 4 Department of Public Health, Our Lady of Fatima University, Valenzuela 838, Philippines; tanfang@foods.ac.cn

**Keywords:** *Lactobacillus plantarum* HFY05, yak yogurt, alcoholic liver injury, mice, expression

## Abstract

Yak yogurt is a type of naturally fermented dairy product prepared by herdsmen in the Qinghai-Tibet Plateau, which is rich in microorganisms. In this study, a strain of *Lactobacillus plantarum* was isolated and identified from yak yogurt in Hongyuan, Sichuan Province and named *Lactobacillus plantarum* HFY05 (LP-HFY05). LP-HFY05 was compared with a common commercial strain of *Lactobacillus delbrueckii subsp*. *bulgaricus* (LDSB). LP-HFY05 showed better anti-artificial gastric acid and bile salt effects than LDSB in in vitro experiments, indicating its potential as a probiotic. In animal experiments, long-term alcohol gavage induced alcoholic liver injury. LP-HFY05 effectively reduced the liver index of mice with liver injury, downregulated the levels of aspartate aminotransferase, alanine aminotransferase, alkaline phosphatase, triglyceride, total cholesterol, blood urea nitrogen, nitric oxide, and MDA and upregulated the levels of albumin, superoxide dismutase (SOD), catalase (CAT), and glutathione peroxidase in the serum of liver-injured mice. LP-HFY05 also reduced the levels of interleukin (IL)-6, IL-12, tumor necrosis factor-alpha, and interferon-gamma in the serum of liver-injured mice. The pathological observations showed that LP-HFY05 reduced the damage to liver cells caused by alcohol. Quantitative polymerase chain reaction and Western blot assays further showed that LP-HFY05 upregulated neuronal nitric oxide synthase, endothelial nitric oxide synthase, manganese-SOD, cuprozinc-SOD, CAT, and inhibitor of κB-α mRNA and protein expression and downregulated the expression of nuclear factor-κB-p65 and inducible nitric oxide synthase in the livers of liver-injured mice. A fecal analysis revealed that LP-HFY05 regulated the microbial content in the intestinal tract of mice with liver injury, increased the content of beneficial bacteria, including *Bacteroides*, *Bifidobacterium*, and *Lactobacillus* and reduced the content of harmful bacteria, including *Firmicutes*, *Actinobacteria*, *Proteobacteria*, and *Enterobacteriaceae*, thus, regulating intestinal microorganisms to protect against liver injury. The effect of LP-HFY05 on liver-injured mice was better than that of LDSB, and the effect was similar to that of silymarin. LP-HFY05 is a high-quality microbial strain with a liver protective effect on experimental mice with alcoholic liver injury.

## 1. Introduction

The Qinghai-Tibet Plateau with its unique geographical location and various climates is an important animal husbandry base for the Zang people. For thousands of years, the Zang people have been eating fermented dairy products prepared by traditional methods. Yak yogurt is a type of fermented milk produced by Zang herdsmen in the Qinghai-Tibet Plateau using ancient and traditional methods [1]. Yak yogurt has high nutritional value and is produced from IV fermented milk, fermented by a naturally selected population of lactic acid bacteria (LAB) and yeast [2]. The yogurt is rich in protein, minerals, lactose, fatty acids, and essential amino acids [3]. The microbial community in yogurt is also very rich, as it is affected by the unique climate, altitude, and technology of the Qinghai-Tibet Plateau. Studies have shown that the beneficial LAB in yak yogurt mainly belong to *Lactobacillus* genus [4]. The LAB isolated from yak yogurt and ghee from different areas in Qinghai, China include *L. casei*, *L. acidophilus*, *L. delbrueckii subsp. bulgaricus*, *L. rhamnosus*, and *L. reuteri* [5].

LAB are the main group of microorganisms involved in the fermentation of traditional fermented foods, such as fermented dairy products [6]. The volatile compounds produced by LAB include small molecules of aldehydes, acids, and esters, which promote the formation of the flavor [7]. LAB are widely distributed in nature. Many studies have shown that they have a clear effect on intestinal diseases. For example, LAB improve digestibility and the utilization rate of food, reduce serum cholesterol, control endotoxins in the body, and inhibit the growth and reproduction of spoilage microorganisms as well as the production of deleterious products in the intestinal tract [8,9]. Other studies have confirmed that LAB have a variety of important physiological functions in the human body, such as maintaining micro-ecological balance, producing nutrition, and stimulating the development of tissues [10]. When probiotic bacteria are low in numbers and harmful bacteria increase, many reactions slow in the body, such as the immune response, inflammatory cells increase, thus, causing a series of metabolic disorders [11,12]. The unique physiology of LAB has allowed them to be widely used in the food, medicine, and other industries [9,13,14].

Alcoholic hepatitis is a liver disease caused by chronic heavy alcohol drinking. It usually presents initially as a fatty liver, which may develop into alcoholic hepatitis, liver fibrosis, and cirrhosis. The main clinical features of alcoholic hepatitis are nausea, vomiting, jaundice, liver swelling, and tenderness pain. It can also cause liver failure and upper-gastrointestinal bleeding [15]. Severe alcoholism leads to extensive necrosis of hepatic cells and liver failure. Alcoholic hepatitis is one of the most common liver diseases and is harmful to health. Alcoholic liver disease (ALD) is mainly an inflammatory reaction directly or indirectly induced by the metabolic processes of ethanol and its derivatives, which is affected by multiple factors, such as oxidative stress, intestinal endotoxins, inflammatory mediators, and a nutritional imbalance, particularly protein-calorie malnutrition [16]. Intestinal endotoxemia caused by damaging intestinal barrier function and endotoxin-activated Kupffer cells play an important role in the occurrence and development of ALD. Intestinal endotoxins combine with plasma proteins, such as lipopolysaccharide-binding protein and soluble CD4, which combine with lipopolysaccharide to form the lipopolysaccharide-lipopolysaccharide-binding protein complex. Lipopolysaccharide significantly increases the transcription and release of inflammatory cytokines, which amplifies the inflammatory effect and stimulates the transformation of stellate cells into fibroblasts, leading to liver fibrosis [17]. In addition, acetaldehyde adducts formed from acetaldehyde and various proteins have strong immunogenicity, and stimulate the production of antibodies in the body, causing immune damage, leading to damage to important proteins, including proteases and DNA [18]. The alcohol factor as the first strike promotes an increase in reactive oxides through oxidative stress and induces the accumulation of liver fat. Under the influence of lipid peroxidation related to oxidative stress and inflammatory cytokines, liver cells with fatty changes are hit a second time, resulting in inflammation, necrosis, and fibrosis [19].

Studies have shown that LAB have an anti-oxidative effect and enhance immunity in vivo [11,12]. The present study tested the ability of *Lactobacillus plantarum* HFY05 (LP-HFY05) to regulate oxidative stress in alcohol-injured mice. This study also observed the effect of LP-HFY05 on the intestinal microbial state of the mice, and clarified the effect of the intestinal microbial state on alcoholic liver injury and the mechanism of LP-HFY05 on alcoholic liver injury.

## 2. Materials and Methods

### 2.1. Isolation and Purification of LAB

One mL of yak yogurt sample from Hongyuan, Sichuan Province was taken and gradient diluted 10 times to with 10^−6^ sterile normal saline. A 100 μL aliquot of the 10^−4^, 10^−5^, and 10^−6^ diluted bacterial liquid samples was spread on a 75 mm microbial culture dish and cultured at 37 °C in sterile MRS agar medium for 24–48 h. The morphology of the bacterial colonies was observed and recorded. Bacterial colonies of different shapes on the plate were selected for streaking. After a 48 h culture at 37 °C, the colonies of different shapes on the plate were picked again for streaking. This process was repeated two to three times until a pure single colony of the same shape was obtained.

### 2.2. Preliminary Identification of the LAB

The pure bacterial colonies on the plate were inoculated in 5 mL MRS liquid medium and cultured at 37 °C for 24 h. One mL of bacteria-containing medium was placed in a sterile centrifuge tube for centrifugation at 4000 r/min for 10 min, and the upper layer was discarded. The bacteria were precipitated and re-suspended in sterile normal saline and examined by Gram staining microscopy. If the Gram-staining microscopy was positive, the bacteria were initially identified as LAB.

### 2.3. Polymerase Chain Reaction (PCR) Amplification and Electrophoretic Detection of Genomic DNA from LAB

The pure suspected target strain was inoculated in MRS broth and cultured at 37 °C for 18–24 h. The DNA was extracted using the bacterial genomic DNA extraction kit. The extracted DNA was amplified by PCR, including 1 μL of the upstream primer 27F (5′-AGA GTT TGA TCC TGGCTC AG-3′), 1 μL of the downstream primer 1495R (5′-CTA CGG CTA CCTTGT TAC GA-3′), 12.5 μL of 2× Taq plus Buffer, and 1 μL of template DNA, and the system was supplemented by adding 25 μL of sterile double distilled H_2_O. Sterile ultrapure water replaced the template DNA as a negative control. The amplification conditions were 94 °C for 5 min, 94 °C for 30 s, 55 °C for 30 s, 29 cycles of 72 °C for 1 min, and 72 °C for 5 min. A 5 μL aliquot of the amplification products was subjected to agarosaccharide gel electrophoresis, which showed that the agarosaccharide concentration was 1.5%. The electrophoresis conditions for detection were 110 V for 45 min. The successfully-detected PCR products were sequenced by 16S rDNA method, and the sequences were compared and analyzed through BLAST (Basic Local Alignment Search Tool) in NCBI [20].

### 2.4. Resistance of LAB to 0.3% Bile Salts

Pig bile salts were added to MRS-THIO culture medium, containing MRS broth with 0.2% sodium thioglycallate, to make a concentration of 0.3%. The medium was sterilized at 121 °C for 15 min. Five mL of the 2% (*v*/*v*) activated strain was inoculated into MRS-THIO medium without bile salts (0.0%) and MRS-THIO medium with 0.3% bile salts in contrast to the blank culture medium (MRS-THIO culture medium without bacteria). After a 24 h culture at 37 °C, the OD_600_ nm value of the culture medium with different concentrations was determined, and the bile salt tolerance of the strain was calculated according to the formula: Bile salt tolerance (%) = (OD_600_ of medium containing 0.3% bile salt − OD_600_ of blank medium)/(OD_600_ of medium containing 0.0% bile salt − OD_600_ of blank medium) × 100 [20].

### 2.5. Ability of LAB to Tolerate Artificial Gastric Juice

The artificial gastric juice was composed of 0.2% NaCl and 0.35% pepsin. The NaCl and pepsin were prepared according to the corresponding mass and volume ratio. The pH of the prepared artificial gastric juice was adjusted to 3.0 with 1 M HCl, and the bacteria were filtered and removed by a 0.22 μm filter membrane for future use. On an ultra-clean workbench, 5 mL of cultivated bacteria-containing medium was drained into a 10 mL sterile centrifuge tube for centrifugation at 3000 r/min for 10 min. The upper layer of the medium was discarded, and the bacteria were collected. A 5 mL aliquot of sterile saline was mixed with the bacteria to make a bacterial suspension. One mL of the bacterial suspension was mixed with 9 mL of pH 3.0 artificial gastric juice. The 1 mL mixture was used as the artificial gastric juice sample processed for 0 h, and the remaining 9 mL of the mixture was put in a water bath shaker at a constant temperature of 37 °C and 150 r/min for 3 h. The 0 and 3 h samples were 10-fold gradient diluted respectively, and the viable count was determined based on the spread-plate method with an appropriate gradient. After a 48-h culture at 37 °C on MRS solid medium, the survival rate was calculated according to the formula: Survival rate (%) = 3 h viable count/0 h viable count × 100 [21]. 

### 2.6. Experiment Strains

The identified LP-HFY05 strain was preserved in the laboratory of the China General Microbiological Culture Collection Center (Beijing, China). The preservation number was CGMCC No. 16635. The *Lactobacillus delbrueckii ssp. Bulgaricus* strain was purchased from the China Center for Type Culture Collection (Preservation number AB200048, Wuhan, China).

### 2.7. Establishment of the Alcoholic Liver Injury Model

Sixty specific pathogen-free six-week-old male Kunming mice were fed for one week to adapt them to the environment and were equally divided into six groups, including the normal group, the model group, the LP-HFY05-L group, the LP-HFY05-H group, the *Lactobacillus delbrueckii ssp. Bulgaricus* (LDSB) group, and the silymarin group (10 mice/group). The mice were given free access to food and water for eight weeks. During the eight weeks, mice in the LP-HFY05-L and LP-HFY05-H groups were given LP-HFY05 by gavage at a concentration of 1.0 × 10^8^ and 1.0 × 10^9^ CFU (colony-forming units)/kg per day, respectively. Mice in the LDSB group received LDSB at a concentration of 1.0 × 10^9^ CFU/kg per day. Mice in the silymarin group were given silymarin at a concentration of 100 mg/kg (b.w) per day. All mice, except those in the normal group, were given 50% alcohol (*v*/*v*) at the concentration of 0.1 mL/10 g per day. After eight weeks, all mice were fasted for 24 h, and blood was taken from the heart and the liver was taken for follow-up experiments. The weight of the liver tissue was measured and the liver index was calculated by the formula [20]. The protocol for these experiments was approved by the Ethics Committee of Chongqing Collaborative Innovation Center for Functional Food (201807006B), Chongqing, China.

### 2.8. Determination of Serum AST, ALT, ALP, TG, TC, BUN, ALB, SOD, NO, CAT, MDA, and GSH-Px Levels 

The plasma was centrifuged at 4000 rpm and 4 °C for 10 min, and the upper layer of serum was taken. The serum levels of aspartate aminotransferase (AST, No. C010-1-1), alanine aminotransferase (ALT, No. C009-1-1), alkaline phosphatase (ALP, No. A059-1-1), triglycerides (TG, No. A110-1-1), total cholesterol (TC, No. A111-1-1), blood urea nitrogen (BUN, No. C013-1-1), albumin (ALB, No. A028-1-1), superoxide dismutase (SOD, No. A001-1-1), nitric oxide (NO, No. A012-1-2), catalase (CAT, No. A007-1-1), malondialdehyde (MDA, No. A003-1-1), and glutathione peroxidase (GSH-Px, No. A005-1-1) were determined in the mice according to the kit instructions (Nanjing Jiancheng Bioengineering Institute, Nanjing, China).

### 2.9. Determination of Serum TNF-α, INF-γ, IL-6, and IL-12 Levels 

Ten μL heparin solution (Nanjing Jiancheng Bioengineering Institute, Nanjing, China) was added to EP tube, and 1 mL plasma of mice was also added to EP tube, and then the plasma was allowed to stand for 30 min, and then the plasma was centrifuged at 4000 rpm and 4 °C for 10 min, and the upper layer of serum was taken. The serum levels of tumor necrosis factor (TNF)-α (No. ab100747), interferon (IFN)-γ (No. ab100689), interleukin (IL)-6 (No. ab100712), and IL-12 (No. ab100699) were determined in the mice according to the enzyme-linked immunosorbent assay kit instructions (Abcam, Cambridge, MA, USA).

### 2.10. Pathological Observations of Liver Tissue 

About 0.5 cm^2^ of liver tissue was removed and fixed in 10% formalin solution for 48 h. The tissues were dehydrated, cleared, waxed, embedded, and sliced for hematoxylin and eosin staining. The morphological changes in the tissue were observed under an optical microscope [22].

### 2.11. qPCR Assay

Mice liver tissue was crushed in a homogenizer containing TRIzol™ Reagent (Invitrogen, Carlsbad, CA, USA) to extract total RNA from the tissue. The extracted total RNA was diluted to 1 μg/μL. A 1 μL aliquot of the diluted total RNA solution was reverse-transcribed using the reverse transcriptase kit method (Thermo Fisher Scientific, Waltham, MA, USA) to obtain the cDNA template. A 1 μL aliquot of the cDNA template, 10 μL of SYBR Green PCR Master Mix (Thermo Fisher Scientific, Waltham, MA, USA), 1 μL each of the upstream and downstream primers (Table 1), and 7 μL of sterile distilled water were mixed and reacted at 95 °C for 60 s, 95 °C for 15 s, 55 °C for 30 s, 72 °C for 35 s, all for 40 cycles. The results were tested at 95 °C for 30 s and at 55 °C for 35 s using StepOnePlus Real-Time PCR System (Thermo Fisher Scientific, Waltham, MA, USA). Relative expression of the genes was calculated using the 2^−ΔΔCt^ method with GAPDH as the internal standard [23].

### 2.12. Western Blot Analysis

A 100 mg portion of a liver tissue sample, 1 mL RIPA, and 10 µL PMSF were added to a homogenizer and activated at 12,000 r/min and 4 °C for 5 min. The mixture was centrifuged at 12,000 r/min and 4 °C for 15 min. The intermediate protein layer solution was taken, and a BCA protein quantitative kit was used to quantify protein. Samples from each group were diluted to 50 µg/mL, and the diluted protein and Sample Buffer were mixed at a ratio of 4:1. The mixture was heated at 100 °C for 5 min and then plunged into an ice bath for 5 min. The mixing acrylamide, resolving buffer, stacking buffer, distilled water, 10% APS, and TEMED were mixed in proportion to make the separation glue and enrichment glue for sodium dodecyl sulfate-polyacrylamide gel electrophoresis (SDS-PAGE), which were injected into a rubber sheet for future use. A prestained protein ladder and the sample were respectively added to the sample holes of the rubber sheet, and the protein-containing SDS-PAGE glue was subjected to vertical gel electrophoresis for 50 min. A PVDF membrane was activated in methanol for 1 min, and the proteins were transferred. After transfer, the membrane was sealed with 1× TBST solution containing 5% skim milk for 1 h. After sealing, the PVDF membrane was washed in 1× TBST and the first antibody was incubated at 25 °C for 2 h. The membrane was washed five times with 1× TBST and the secondary antibody was incubated at 25 °C for 1 h. Finally, the PVDF membrane was sprinkled with Supersignal West Pico PLUS and placed in the iBright FL1000 for observation [24].

### 2.13. Detection of Intestinal Microbiota in Feces

DNA of the mice stool microbes was extracted with the QIAamp DNA Stool Mini Kit (Qiagen, Valencia, CA, USA). Then, qPCR was used to detect changes in the content of *Firmicutes*, *Bacteroides*, *Actinomycetes*, *Proteobacteria*, *Bifidobacterium*, *Lactobacillus*, and *Enterococcus* in mice stool from each group. The primer sequences are shown in Table 2. And the remaining experimental steps are the same as those in 2.11. Since the Ct value was linear with the logarithm of the initial number of copies of the template, the standard curve was made by using the standard universal primer of the number of copies. The Ct value of the sample is compared with the curve of the standard, and the copy number of the sample can be calculated from the standard curve. 

### 2.14. Statistical Analysis

The serum and tissue detection assays were performed in three parallel experiments and the average was recorded. Data were analyzed using SPSS software (SPSS Inc., Chicago, IL, USA. One-way analysis of variance was used to detect significant differences between the groups at the *p* < 0.05 level.

## 3. Results

### 3.1. Colony Morphology and LAB Cell Morphology 

**Figure 1:**
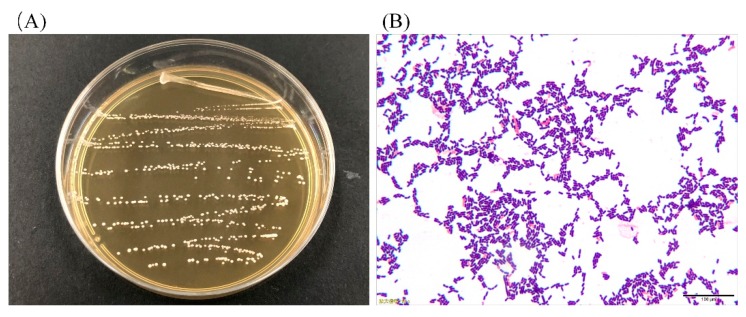
Colony morphology (**A**) and cell morphology (**B**) of the strain.

As shown in Figure 1A, the colonies were mostly white or milky white in color, with a round shape, neat edges, and a moist and smooth surface. The cell morphology of the strain under 100× oil immersion was long and short rods, and no budding reproduction was detected (Figure 1B). As a result, it can be preliminarily judged that the isolated strain was a Gram-positive bacillus.

### 3.2. Analysis of 16S rDNA of the LAB

**Figure 2:**
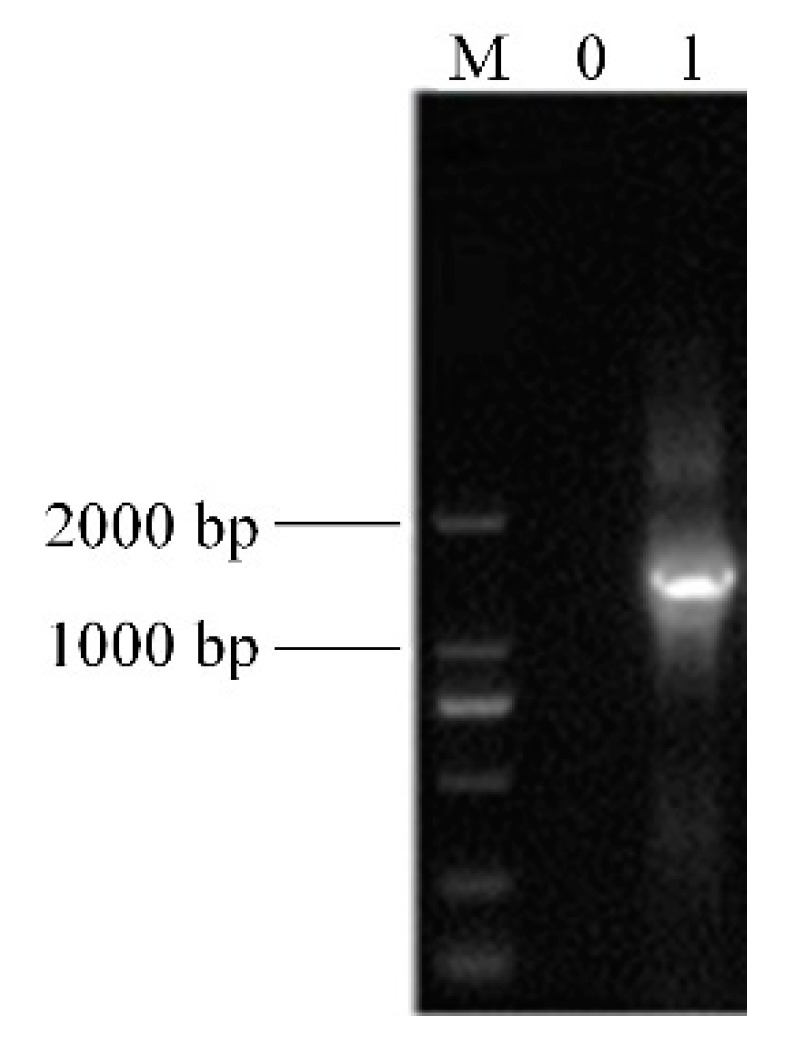
Agarose gel electrophoresis of the 16S r DNA-PCR products from the strain. Note: M, 2000 bp DNA ladder; 0, negative control group; 1, strain.

The extracted DNA was amplified by PCR, the 16S rDNA amplification product was detected by 1.5% agarose gel electrophoresis and the results are shown in Figure 2. With sterile ultra-pure water as a negative control, the lane showed no bands, indicating that the PCR amplification process was not contaminated. The length of the lane 2-specific amplified fragment was about 1500 bp, which was in accordance with the expected length of the amplified fragment. A sequence was detected using 16S rDNA method, compared, and analyzed by the BLAST program and showed that the homology between the strain and the known *Lactobacillus* in the GenBank database was 99% (Gen Bank No. MN368557.1), so it belonged to *Lactobacillus* and was named *Lactobacillus plantarum* HFY05.

### 3.3. Resistance of LAB to Artificial Gastric Juice and Bile Salts

Table 3 shows that the survival rate of LP-HFY05 in pH 3.0 artificial gastric juice was close to 70%. The survival rate in 0.3% bile salts reached 20.77%, indicating a strong tolerance to gastric acid and bile salts, so this was a strain with probiotic potential.

### 3.4. Mice Liver Indices 

**Figure 3:**
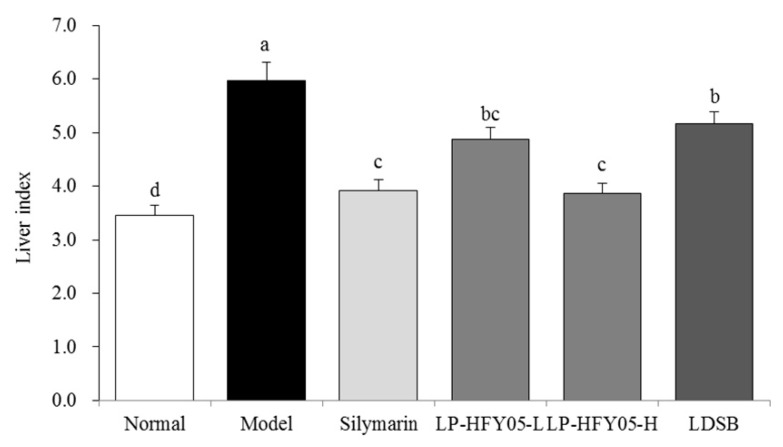
Liver index values of mice in each group (*N* = 10). Values are mean ± standard deviation (*N* = 10/group). Sample data in each group come from a normal distribution. The difference in variance between the two groups was significant (*p* < 0.05). ^a–e^ Mean values with different letters over the bar are significantly different (*p* < 0.05) according to Tukey’s honestly significantly different test. Silymarin: Mice treated with silymarin (100 mg/kg), LP-HFY05-L: Mice treated with a low concentration of *Lactobacillus plantarum* HFY05 (1.0 × 10^8^ CFU/kg), LP-HFY05-H: Mice treated with a high concentration of *Lactobacillus plantarum* HFY05 (1.0 × 10^9^ CFU/kg), LDSB: Mice treated with *Lactobacillus delbrueckii subsp. bulgaricus* (1.0 × 10^9^ CFU/kg).

As shown in Figure 3, the liver index of the model group was the highest (5.97 ± 0.34), while the liver index of the normal group was the lowest (3.46 ± 0.18). Because of the action of LP-HFY05-H, the liver index of injured mice decreased to 3.87 ± 0.19, which was lower than that of the LP-HFY05-L (4.88 ± 0.22) and LDSB groups (5.16 ± 0.23) but close to that of the silymarin group (3.92 ± 0.21). LP-HFY05 effectively reduced the liver index of liver-injured mice, and the effect was enhanced with an increase of concentration.

### 3.5. Serum Levels of AST, ALT, ALP, TG, TC, BUN, and ALB in the Mice

As shown in Table 4, the serum ALB level in the model group mice was lower than that in the normal group mice, but the other indicators (AST, ALT, ALP, TG, TC, and BUN) were all higher than those in the normal group mice. LP-HFY05-H reduced the levels of AST, ALT, ALP, TG, TC, and BUN but increased the ALB level in the serum of liver-injured mice, and the effect was significantly better than that of LDSB (*p* < 0.05), which was close to that of silymarin.

### 3.6. Serum Levels of SOD, NO, CAT, MDA, and GSH-Px in the Mice

As shown in Table 5, the activities of SOD, CAT, and GSH-Px were significantly higher in the normal group mice than those in the other groups (*p* < 0.05), while the contents of NO and MDA were significantly lower than those in the other groups (*p* < 0.05). Liver injury caused by alcohol (model group) decreased the activities of SOD, CAT, and GSH-Px in the serum of mice and increased the contents of NO and MDA. LP-HFY05 effectively inhibited the decrease in oxidation-related enzymes (SOD, CAT, and GSH-Px) in serum caused by liver injury and increased the NO and MDA contents. A high concentration of LP-HFY05 had a better effect, than that of LDSB and was similar to silymarin.

### 3.7. Serum Levels of IL-6, IL-12, TNF-α, and IFN-γ in Mice

Table 6 shows that the levels of IL-6, IL-12, TNF-α, and IFN-γ were significantly lower in the normal group of mice than those in the other groups (*p* < 0.05), while the levels of these cytokines were significantly higher in the model group than those in the other groups (*p* < 0.05). LP-HFY05 effectively decreased the levels of IL-6, IL-12, TNF-α, and IFN-γ in the serum of liver-injured mice, and the effect of high concentration LP-HFY05 (LP-HFY05-H) was better than that of a low concentration of LP-HFY05 (LP-HFY05-H). LP-HFY05 had a stronger effect on serum cytokine levels in the liver-injured mice than LDSB, which was close to silymarin.

### 3.8. Pathological Observations of Mice Liver Tissue 

**Figure 4:**
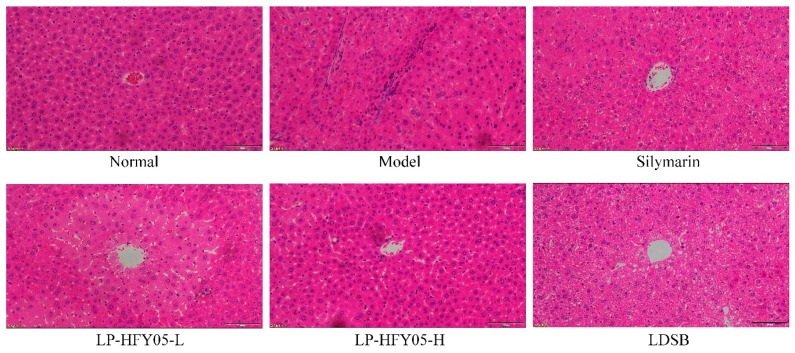
Hematoxylin and eosin pathological observations of hepatic tissues from mice. Magnification 100× (scale bar: 100 μm). Silymarin: Mice treated with silymarin (100 mg/kg), LP-HFY05-L: Mice treated with a low concentration of *Lactobacillus plantarum* HFY05 (1.0 × 10^8^ CFU/kg), LP-HFY05-H: Mice treated with a high concentration of *Lactobacillus plantarum* HFY05 (1.0 × 10^9^ CFU/kg), LDSB: Mice treated with *Lactobacillus delbrueckii subsp. bulgaricus* (1.0 × 10^9^ CFU/kg).

As shown in Figure 4, the structure of the liver tissue cells in the normal group was normal, and the liver cells were radially distributed and centered around a central vein. In the model group, the liver cells were not arranged evenly, the central vein was irregular, the cell structure was destroyed, and a large number of cells were necrotic. LP-HFY05, silymarin, and LDSB reduced the necrosis and destruction of liver tissue morphology caused by alcohol in liver cells, and the effect of a high concentration LP-HFY05 (LP-HFY05-H) was the best, which was close to that of the drug silymarin and better than that of LDSB.

### 3.9. nNOS, eNOS, and iNOS mRNA and Protein Expression in Mouse Liver Tissue

**Figure 5:**
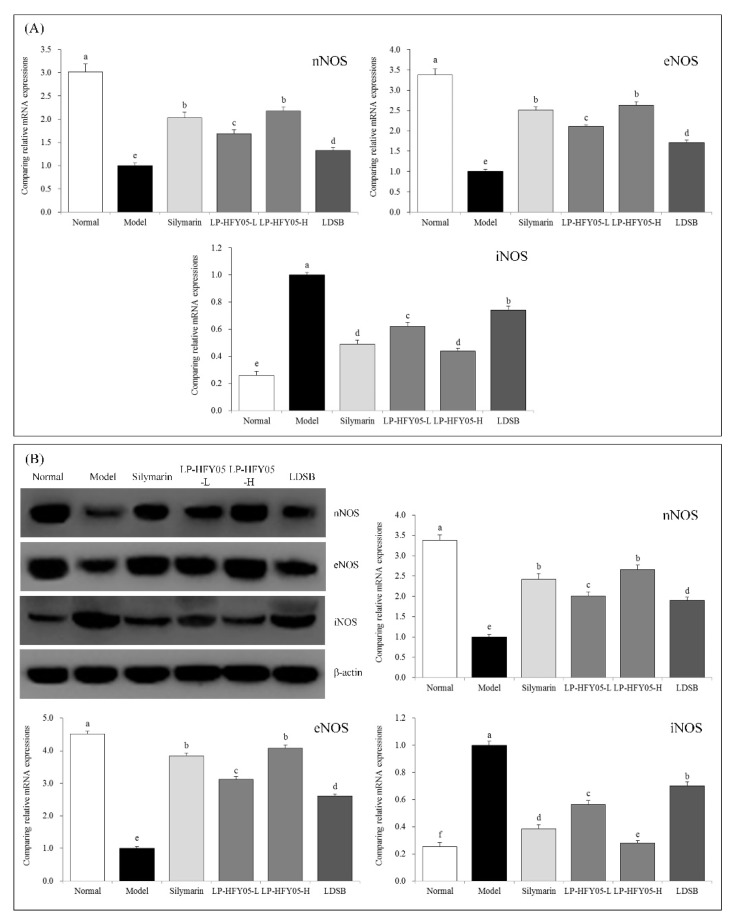
Neuronal nitric oxide synthase (nNOS), endothelial nitric oxide synthase (eNOS), and inducible nitric oxide synthase (iNOS) mRNA (**A**) and protein (**B**) expression in hepatic tissues of the mice. Sample data in each group came from a normal distribution. The difference in variance between the two groups was significant (*p* < 0.05). ^a–f^ Mean values with different letters in the bar are significantly different (*p* < 0.05) according to Tukey’s honestly significant different test. Silymarin: Mice treated with silymarin (100 mg/kg), LP-HFY05-L: Mice treated with a low concentration of *Lactobacillus plantarum* HFY05 (1.0 × 10^8^ CFU/kg), LP-HFY05-H: Mice treated with a high concentration of *Lactobacillus plantarum* HFY05 (1.0 × 10^9^ CFU/kg), LDSB: Mice treated with *Lactobacillus delbrueckii subsp. bulgaricus* (1.0 × 10^9^ CFU/kg).

As shown in Figure 5, the expression of neuronal nitric oxide synthase (nNOS) and endothelial nitric oxide synthase (eNOS) mRNA and protein in liver tissue of mice in the normal group was the strongest, while inducible nitric oxide synthase (iNOS) expression was the weakest. Alcohol weakened the expression of nNOS and eNOS in liver tissue and strengthened the expression of iNOS. LP-HFY05 inhibited the increased expression of iNOS and decreased the expression of nNOS and eNOS. The effect of a high concentration of LP-HFY05 was slightly stronger than that of silymarin and significantly stronger than that of LDSB (*p* < 0.05).

### 3.10. Cu/Zn-SOD, Mn-SOD, and CAT mRNA and Protein Expression in Mouse Liver Tissue

**Figure 6:**
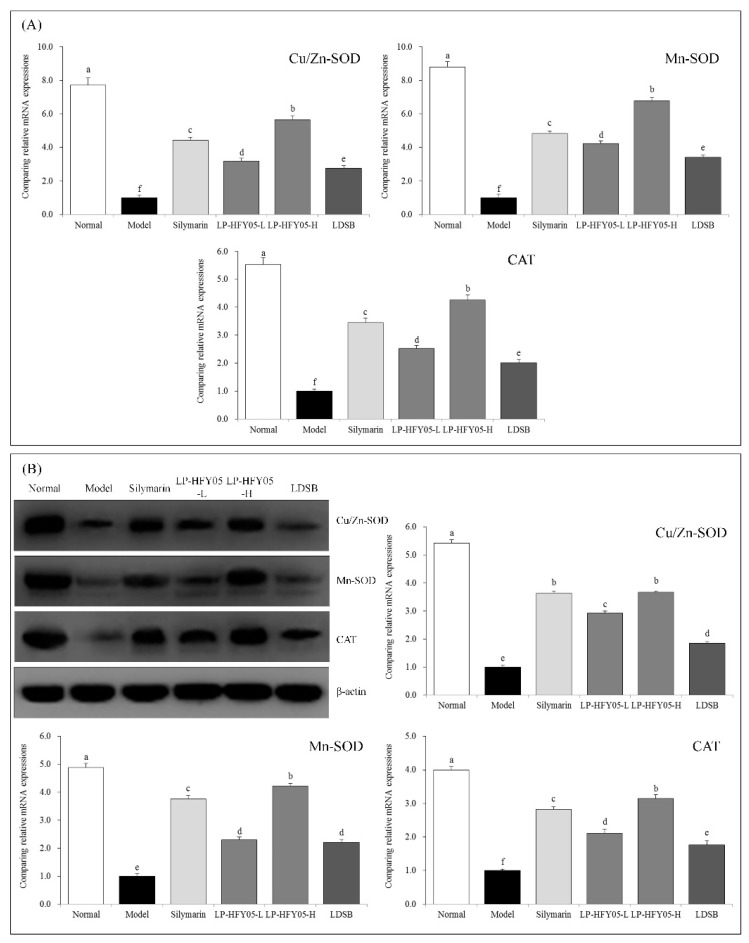
The Cu/Zn-superoxide dismutase (SOD), Mn-SOD, and catalase (CAT) mRNA (**A**) and protein (**B**) expression in hepatic tissues of mice. Sample data in each group come from a normal distribution. The difference in variance between the two groups was significant (*p* < 0.05). ^a–f^ Mean values with different letters in the bar are significantly different (*p* < 0.05) according to Tukey’s honestly significantly different test. Silymarin: Mice treated with silymarin (100 mg/kg), LP-HFY05-L: Mice treated with a low concentration of *Lactobacillus plantarum* HFY05 (1.0 × 10^8^ CFU/kg), LP-HFY05-H: Mice treated with a high concentration of *Lactobacillus plantarum* HFY05 (1.0 × 10^9^ CFU/kg), LDSB: Mice treated with *Lactobacillus delbrueckii subsp. bulgaricus* (1.0 × 10^9^ CFU/kg).

As shown in Figure 6, Mn-SOD, Cu/Zn-SOD, and CAT mRNA and protein expression in the livers of mice in the normal group was the strongest, while that in the model group was the weakest. Silymarin administered by gavage significantly upregulated the expression of Mn-SOD, Gu/Zn-SOD, and CAT in liver of liver-injured mice, while LP-HFY05 upregulated expression of these molecules, and a high concentration of LP-HFY05 had a more obvious effect (*p* < 0.05), which was better than the low concentrations of LP-HFY05 and LDSB, but was not significantly different from silymarin (*p* > 0.05).

### 3.11. NF-κB p65 and IκB-α mRNA and Protein Expression in Mouse Liver Tissue

**Figure 7:**
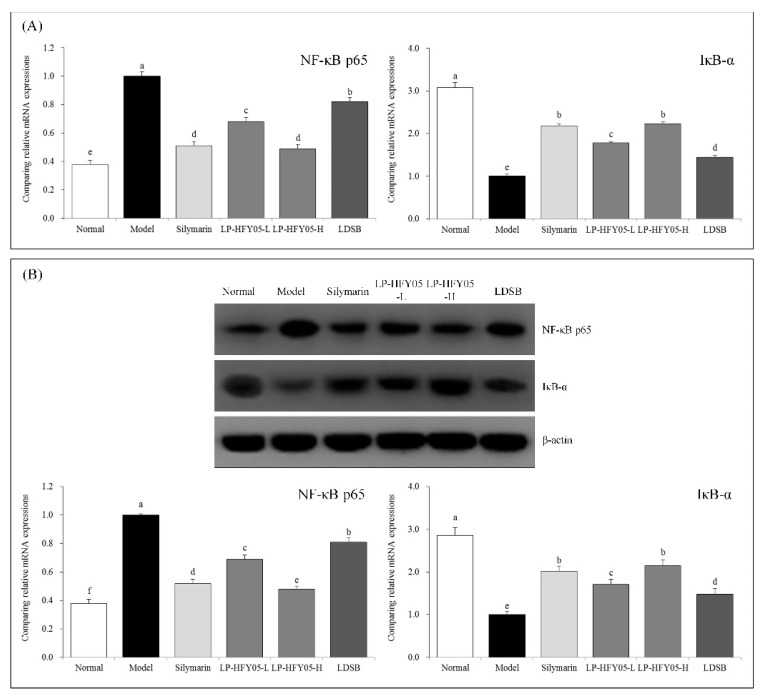
Nuclear factor (NF)κB-p65 and inhibitor of kappa beta (IκB)-α mRNA (**A**) and protein (**B**) expression in hepatic tissues of mice. Sample data in each group come from a normal distribution. The difference in variance between the two groups was significant (*p* < 0.05). ^a–f^ Mean values with different letters in the bar are significantly different (*p* < 0.05) according to Tukey’s honestly significantly different test. Silymarin: Mice treated with silymarin (100 mg/kg), LP-HFY05-L: Mice treated with a low concentration of *Lactobacillus plantarum* HFY05 (1.0 × 10^8^ CFU/kg), LP-HFY05-H: Mice treated with a high concentration of *Lactobacillus plantarum* HFY05 (1.0 × 10^9^ CFU/kg), LDSB: Mice treated with *Lactobacillus delbrueckii subsp. bulgaricus* (1.0 × 10^9^ CFU/kg).

According to Figure 7, after alcohol-induced liver injury, the expression of nuclear factor kappa beta (NF-κB) p65 in the liver of the mice was significantly stronger in the model group than that in the other groups (*p* < 0.05), while IκB-α expression was significantly weaker than that in the other groups (*p* < 0.05). The expression intensity trend of NF-κB p65 and IκB-α in the liver of mice in the normal group was opposite to that of mice in the model group. Compared with the model group, silymarin and LP-HFY05 downregulated the expression of NF-κB p65 and upregulated expression of IκB-α in the liver. A high concentration LP-HFY05 downregulated the expression of NF-κB p65 and upregulated expression of IκB-α in the liver, which was stronger than LP-HFY05-L and LDSB and similar to silymarin.

### 3.12. Intestinal Fecal Microbiota of Mice

Table 7 shows that *Bacteroides*, *Bifidobacterium*, and *Lactobacillus* were the lowest in the stool of mice in the model group, while *Firmicutes*, *Actinobacteria*, *Proteobacteria*, and *Enterobacteriaceae* were the highest. LP-HFY05 given by gavage reduced *Firmicutes*, *Actinobacteria*, *Proteobacteria*, and *Enterobacteriaceae* contents and increased *Bacteroides*, *Bifidobacterium*, and *Lactobacillus* contents. LP-HFY05 better intervened in the intestinal microbiota of mice than LDSB at the same concentration. *Lactobacillus* in the intestinal tract was even higher than that of mice in the normal group after LP-HFY05 was given by gavage. In addition, the changes in the microbiota of the intestinal tract were smaller after silymarin was given by gavage, than that after LP-HFY05 and LDSB were given by gavage. Therefore, the regulatory effect of LP-HFY05 on alcoholic liver injury may come from its regulatory effect on intestinal microorganisms, which is different from the mechanism of silymarin.

## 4. Discussion

The liver plays an important role in maintaining balance in the body and regulating the body’s state. It participates in many physiological processes, such as growth and development, fighting disease, and supplying energy. The liver is also the main metabolic site of carbohydrates, proteins, and lipids and it secretes bile and stores vitamins. The liver is the largest detoxification organ in body. Therefore, the status of the liver is an important factor ensuring health. With the improvements in lifestyle and dietary structure and increased alcohol production, the proportion of the drinking population is on the rise. The incidence of alcohol-related diseases is also increasing annually. Chronic alcoholism leads to alcoholic diseases, such as alcoholic hepatitis, fatty liver, and cirrhosis [25]. As a classic liver-protection drug, silymarin has been used for a long time to treat various kinds of liver injuries, as it has anti-oxidation, anti-inflammation, immune regulatory, and cell regeneration promoting effects [26]. In this study, silymarin was used as a positive control drug. Liver weight and the liver index, as an evaluation of the degree of carbon tetrachloride-induced liver injury, have been applied in research. High liver weight quality and the liver index has been used to assess liver injury [27]. We showed that LP-HFY05 downregulated liver weight quality and the liver index in liver-injured mice, and the effect of liver injury treatment with LP-HFY05 was similar to that of silymarin.

AST mainly exists in the cytoplasm and mitochondria of hepatocytes, while ALT mainly exists in the cytoplasm, so necrosis of hepatocytes increases ALT and AST in the body [28]. After lipid peroxidation of liver tissues, the permeability of the liver membranes change, and the ALP level in liver cells increases sharply [29]. Liver injury can lead to the transfer of fatty acids to the liver, resulting in increased TG content in the liver. TG and TC also reflect lipid peroxidation in the liver, and lipid peroxidation increases the level of TG and TC in the body [30]. The influence of liver injury on liver function leads to impaired renal function and increased protein metabolic products and BUN in the blood. Liver injury also affects the synthesis, transport, and release of ALB in the body and decreases blood ALB content [31]. The present study showed that LP-HFY05 almost restored these blood indices to a normal state, and the effect was similar to that of silymarin.

Long-term drinking causes oxidation in the body, and the body defends against oxidative damage using non-enzymatic and enzymatic antioxidants. Enhancing SOD, CAT, and GSH-Px activities is the main mechanism of enzymatic antioxidation in the body [32]. SOD catalyzes and removes superoxide free radicals. CAT and SOD have a synergistic effect on the removal of free radicals [33]. CAT removes H_2_O_2_ in the body, thus, inhibiting oxidative stress reactions and alleviating the oxidation caused by alcohol to lessen liver injury [34]. GSH-Px is an enzyme that catalyzes the decomposition of H_2_O_2_. Catalyzing the reduction reaction of reduced glutathione to H_2_O_2_ protects cell membranes from damage [35]. MDA is a lipid peroxidation metabolite that accumulates in large quantities in the body after liver injury [36]. An increase in NO and its oxidation products leads to damage to phospholipids and proteins on the surface of cells, thereby promoting inflammatory exudation of tissues [37]. At the same time, NO reacts with superoxide anions to form ONOO- superoxide nitrite anions and intensify the oxidative stress reaction, further exacerbating liver injury due to cytotoxicity [38]. LP-HFY05 upregulated the activities of SOD, CAT, and GSH-Px and downregulated the serum levels of MDA and NO in liver-injured mice, thereby intervening against liver injury.

Alcohol leads to oxidation in the body and promotes an inflammatory reaction in the liver, which significantly increases serum levels of IL-6, IL-12, TNF-α, and IFN-γ in mice [39]. IL-6 is secreted by Th2 cells and is involved in the humoral immune response. The increased level of Th2 cells in the body causes visceral functional damage [40]. IL-6 promotes the differentiation and proliferation of T lymphocytes, promotes the production of antibodies, changes the activity of intracellular G cells, and upregulates the function of neutrophils, thus, enhancing the inflammatory response of the body [41]. IL-12 is an activator of NK cells. Excess apoptosis of liver cells and an excessive immune response in liver injury intensify the injury, which is related to the increased killing function of CD8+ T cells by IL-12 [42]. The combination of TNF-α and TNF-αR1 on the liver cell membrane leads to the transformation of double-stranded genomic DNA into oligonucleotide fragments and promotes apoptosis of stem cells. In addition, TNF-α aggravates inflammation and liver tissue injury in the body by activating NF-κB [43]. IFN-γ is a pro-inflammatory cytokine that enhances the sensitivity of liver cells to TNF-α, making liver cells more easily damaged [44]. The oxidative stress state after liver injury causes an imbalance in inflammatory factors, including TNF-α and IL-6 in the body, leading to an increase in TNF-α and IL-6 in the liver [45]. LP-HFY05 reduced liver injury by regulating the levels of IL-6, IL-12, TNF-α, and IFN-γ in the serum of liver-injured mice, which was a similar effect as silymarin.

nNOS protects nerve cells. nNOS is abundant in endocrine cells and plays a positive role in repairing damaged tissues [46]. The expression of eNOS in tissues is relatively stable. NO produced by eNOS promotes the repair of liver tissues. eNOS also promotes the regeneration of blood vessels in liver tissues [47]. NOS, as the rate-limiting enzyme in the synthesis of NO, exists in large quantities in normal tissues. After activation, NO activity can last for a long time, thus releasing a large amount of NO. A low concentration of NO inhibits gene mutations and enhances the body’s defense ability. However, excessive NO leads to maladjustment in the control of gene mutations, stimulates gene mutations, and causes tissue lesions [48]. iNOS expression is high in inflammatory parts of the body, and its activation produces a large number of inflammatory mediators, leading to aggravation of liver injury [49]. LP-HFY05 upregulated the expression of nNOS and eNOS and downregulated the expression of iNOS, thus, controlling inflammation.

Mn-SOD and Gu/Zn-SOD are isomers of SOD in the body. Mn-SOD is a SOD-free radical scavenger with Mn^4+^ as the active center of mitochondria. Cu/Zn-SOD is another SOD-free radical scavenger with Cu^2+^ and Zn^2+^ as the active center in the cytoplasm [48]. Mitochondria are most abundant in the liver and heart. Mn-SOD activity decreases significantly after liver injury from alcohol, and this study obtained the same result [50]. Cu/Zn-SOD removes the toxic effect caused by O^2−•^ in the body and protects visceral tissues [51]. Studies have shown that alcohol causes oxidative stress reactions and produces a large amount of free radicals. Mn-SOD and Cu/Zn-SOD inhibit free radicals, thus, preventing liver injury [52]. LP-HFY05 improved Mn-SOD and Cu/Zn-SOD mRNA and protein expression in liver tissue, thereby promoting repair of liver injury.

NF-κB interferes with the expression of related genes by combining with target proteins, thereby affecting the growth and differentiation of cells, inflammatory reactions, and related immune reactions, so it is a key transcriptional inducing factor in vivo. NF-κB also regulates the production of relevant inflammatory transmitters, such as TNF-α and IL-6, which are downstream molecules regulated by NF-κB [53]. IκB has many forms, including IκB-α, IκB-β, and IκB-γ, which combine with NF-κB in an inactive state to form an inactive trimer. When the liver is injured, pathogenic molecules, such as TNF-α, activate protein kinase C. After IκB phosphorylation, it is degraded and exposed to NF-κB for activation and then the transcriptional program starts [54]. LP-HFY05 enhanced NF-κB mRNA and protein expression and reduced IκB-α mRNA and protein expression in liver tissues, thus, reducing liver injury.

The current in-depth research showed that an imbalance in intestinal microbiota played an important role in the occurrence and development of inflammation. Imbalance in the intestinal microbiota can lead to the production of harmful bacteria, particularly flagellated Gram-negative bacteria, resulting in excessive production of lipopolysaccharide (LPS). LPS enters the blood circulation through damaged intestinal epithelial cells and causes low-grade chronic inflammation throughout the body. Inflammatory infiltration of the liver occurs when liver function is marginal, and the body’s metabolism is impaired [55]. Improving an intestinal microbiota disorder reduces the number of harmful bacteria, particularly *Firmicutes*, and increases the number of beneficial bacteria, particularly *Bacteroides*. Studies have shown that because of chronic alcohol intake, the amount of aerobes in the small intestine of mice is about 10 times more than that in the small intestine of mice in the control group, with *Staphylococcus aureus* increasing significantly. The number of *Lactobacillus* and the proportion of aerobes decrease, showing that long-term drinking leads to the overgrowth of intestinal bacteria, weakens the protective effect of LAB on the intestinal mucosa, and increases the pathogenicity of aerobic bacteria, such as *Escherichia coli* [56]. Compared with a normal state, anaerobic bacteria, such as *Bifidobacterium*, significantly decrease in patients with liver injury, while opportunistic pathogens, such as *Enterococcus* and *Enterobacter*, increase significantly, and the degree of change is related to liver function grading. The more severe the lesion, the more *Enterococcus*, and *Enterobacter* are found [57]. Alcohol intake can lead to liver dysfunction, decrease the secretion of bile, and damage the local immune defense system of the gastrointestinal tract, leading to excessive growth of intestinal bacteria, particularly Gram-negative bacteria and an imbalance in the intestinal microbiota [58]. Alcohol increases the number of intestinal *Proteus* and Gram-negative bacteria, thereby decreasing the number of *Bifidobacterium*. Because *Proteus* is considered to be an important bacterium to activate the innate immune system, the increase in *Proteus* activates the immune system, which promotes a chronic inflammatory response in the liver [59]. In addition, alcohol intake can increase the numbers of *Proteobacteria* and *Actinobacteria* in the intestinal tract, which promotes more growth of pathogenic bacteria, leading to an intestinal microbiota disorder. The close connection between the decrease in the intestinal mucosa cells and the increase in permeability reduces the barrier function of the intestinal mucosa and causes translocation of a large number of endotoxins into the portal system. Large numbers of liver Kupffer cells are collected and a series of cell inflammatory factors, such as TNF, IL, and arachidonic acid, are released to attack the liver. Probiotic supplements decrease the number of Gram-negative bacteria and improve the changes in intestinal microbiota caused by alcohol, thereby relieving liver injury [60]. The results of this study also show that LP-HFY05 regulated the microecology in the intestinal tract of mice by increasing the contents of *Bacteroides*, *Bifidobacterium*, and *Lactobacillus* and decreasing the contents of harmful bacteria, such as *Firmicutes*, *Actinobacteria*, *Proteobacteria,* and *Enterobacteriaceae*. The reason may be that LP-HFY05 most probably is able to produce exopolysaccharides and for this reason can adhere to the epithelial cells [61], LP-HFY05 directly or indirectly affected intestinal epithelial cells, enhanced intestinal barrier function, and modulated the mucosal immune system. The results show that LP-HFY05 could reduce the number of harmful bacteria, including Gram-negative bacteria, so as to avoid damaging the liver by toxic substances produced by harmful bacteria. Thus, LP-HFY05 may be a good probiotic to alleviate alcoholic liver injury.

## 5. Conclusions

This study showed that LP-HFY05 reduced the liver index of liver-injured mice and downregulated the levels of AST, ALT, ALP, TG, TC, BUN, NO, and MDA and upregulated the levels of ALB, SOD, CAT, and GSH-Px in the serum of liver-injured mice. LP-HFY05 also downregulated the serum levels of IL-6, IL-12, TNF-α, and IFN-γ in the liver-injured mice. qPCR and Western blot experiments further confirmed that LP-HFY05 effectively upregulated nNOS, eNOS, Mn-SOD, Cu/Zn-SOD, CAT, and IκB-α mRNA and protein expression and downregulated NF-κB-p65 and iNOS expression in the liver tissues of liver-injured mice. Further observations determined that LP-HFY05 regulated the intestinal microecology of the alcoholic liver-injured mice, increased the number of beneficial bacteria, and decreased the number of harmful bacteria, thus, playing a role alleviating liver injury LP-HFY05 had an interventional effect on alcoholic liver injury, and the effect was close to that of the drug silymarin. However, the effect of LP-HFY05 on human intestinal microecology and human liver tissue still needs further research. LP-HFY05 is expected to be used as a probiotic after a human study.

## Figures and Tables

**Table 1:** Sequences of the primers used for the mice liver tissue.

Gene Name	Sequence
*nNOS*	Forward: 5′-GAATACCAGCCTGATCCATGGAA-3′
	Reverse: 5′-TCCTCCAGGAGGGTGTCCACCGCATG-3′
*eNOS*	Forward: 5′-TCAGCCATCACAGTGTTCCC-3′
	Reverse: 5′-ATAGCCCGCATAGCGTATCAG-3′
*iNOS*	Forward: 5′-GTTCTCAGCCCAACAATACAAGA-3′
	Reverse: 5′-GTGGACGGGTCGATGTCAC-3′
*Cu*/*Zn*–*SOD*	Forward: 5′–AACCAGTTGTGTTGTCAGGAC–3′
	Reverse: 5′–CCACCATGTTTCTTAGAGTGAGG–3′
*Mn*–*SOD*	Forward: 5′-CAGACCTGCCTTACGACTATGG-3′
	Reverse: 5′-CTCGGTGGCGTTGAGATTGTT-3′
*CAT*	Forward: 5′-GGAGGCGGGAACCCAATAG-3′
	Reverse: 5′-GTGTGCCATCTCGTCAGTGAA-3′
*IκB*-*α*	Forward: 5′-CGCGGGATGGCCTCAAGAAGGA-3′
	Reverse: 5′-GCCAAGTGCAGGAACGAGTCT-3′
*NF*-*κB p65*	Forward: 5′-GAGGCACGAGGCTCCTTTTCT-3′
	Reverse: 5′-GTAGCTGCATGGAGACTCGAACA-3′
*GAPDH*	Forward: 5′-AGGTCGGTGTGAACGGATTTG-3′
	Reverse: 5′-GGGGTCGTTGATGGCAACA-3′

*nNOS*: Neuronal nitric oxide synthase, *eNOS*: Endothelial nitric oxide synthase, *iNOS*: Inducible nitric oxide synthase, *Cu/Zn–SOD*: Cuprozinc–superoxide dismutase, *Mn–SOD*: Manganese superoxide dismutase, *CAT*: Catalase, *NF-κB p65*: Nuclear factor kappa-B p65, *IκB-α*: Inhibitor of NF-κB alpha, *GAPDH*: Glyceraldehyde-3-phosphate dehydrogenase.

**Table 2:** Sequences of primers used for intestinal microbiota in mice feces.

Gene Name	Sequence
*Firmicutes*	Forward: 5′-GGAGYATGTGGTTTAATTCGAAGCA-3′
	Reverse: 5′-AGCTGACGACAACCARGCAC-3′
*Bacteroides*	Forward: 5′-GGARCATGTGGTTTAATTCGATGAT-3′
	Reverse: 5′-AGCTGACGACAACCATGCAG-3′
*Actinobacteria*	Forward: 5′-TACGGCCGCAAGGCTA-3′
	Reverse: 5′-TARTCCCCACCTTCCTCCG-3′
*Proteobacteria*	Forward: 5′–CATGACGTTACCCGCAGAAGAA–3′
	Reverse: 5′–CTCTACGAGACTCAAGCTTGC–3′
*Bifidobacterium*	Forward: 5′-TCGCGTC(C/T)GGTGTGAAAG-3′
	Reverse: 5′-CCACATCCAGC(A/G)TCCAC-3′
*Lactobacillus*	Forward: 5′-AGCAGTAGGGAATCTTCCA-3′
	Reverse: 5′-CACCGCTACACATGGAG-3′
*Enterobacteriaceae*	Forward: 5′-CATTGACGTTACCCGCAGAAGAAGC-3′
	Reverse: 5′-CTCTACGAGACTCAAGCTTGC-3′
Universal primer	Forward: 5′-ACTCCTACGGGAGGCAGCAG-3′
	Reverse: 5′-ATTACCGCGGCTGCTGG-3′

**Table 3:** Resistance of LAB to artificial gastric juice and bile salts.

Treatment	Survival Rate in Artificial Gastric Juice at pH 3.0 (%)	Survival Rate in 0.3% Bile Salt (%)
LP-HFY05	69.38 ± 4.62	20.77 ± 1.26
LDSB	34.57 ± 2.29	7.03 ± 0.38

LDSB: *Lactobacillus delbrueckii subsp. bulgaricus*, LP-HFY05: *Lactobacillus plantarum* HFY05.

**Table 4:** Levels of AST, ALT, ALP, TG, TC, BUN and ALB in mice serum (*N* = 10).

Group	ALT (U/L)	AST (U/L)	ALP (K-A)	TG (mmol/L)	TC (mmol/L)	BUN (mg/dL)	ALB (g/dL)
Normal	15.82 ± 1.62 ^e^	11.10 ± 0.47 ^e^	6.88 ± 0.71 ^d^	0.42 ± 0.04 ^e^	1.42 ± 0.20 ^e^	20.38 ± 2.44 ^e^	3.83 ± 0.12 ^a^
Model	67.32 ± 3.23 ^a^	53.69 ± 2.91 ^a^	15.37 ± 2.42 ^a^	1.81 ± 0.13 ^a^	5.71 ± 0.48 ^a^	47.58 ± 3.91 ^a^	2.56 ± 0.13 ^e^
Silymarin	30.86 ± 1.79 ^d^	20.36 ± 1.21 ^d^	9.12 ± 1.05 ^c^	0.72 ± 0.07 ^d^	2.43 ± 0.27 ^d^	30.18 ± 2.23 ^d^	3.22 ± 0.08 ^b^
LP-HFY05-L	46.17 ± 2.88 ^c^	31.59 ± 1.93 ^c^	12.30 ± 1.12 ^b^	1.25 ± 0.10 ^c^	3.49 ± 0.31 ^c^	35.25 ± 1.98 ^c^	2.96 ± 0.10 ^c^
LP-HFY05-H	28.97 ± 2.15 ^d^	19.17 ± 1.33 ^d^	9.01 ± 0.82 ^c^	0.64 ± 0.06 ^d^	2.29 ± 0.25 ^d^	28.97 ± 2.12 ^d^	3.31 ± 0.10 ^b^
LDSB	53.17 ± 2.08 ^b^	35.07 ± 1.60 ^b^	13.57 ± 1.18 ^b^	1.49 ± 0.09 ^b^	4.12 ± 0.26 ^b^	40.39 ± 2.07 ^b^	2.71 ± 0.11 ^d^

Values are mean ± standard deviation (*N* = 10/group). Sample data in each group come from a normal distribution. The difference in variance between the two groups was significant (*p* < 0.05). ^a–e^ Mean values with different letters over the same column are significantly different (*p* < 0.05) according to Tukey’s honestly significant different test. Silymarin: Mice treated with silymarin (100 mg/kg), LP-HFY05-L: Mice treated with a low concentration of *Lactobacillus plantarum* HFY05 (1.0 × 10^8^ CFU/kg), LP-HFY05-H: Mice treated with a high concentration of *Lactobacillus plantarum* HFY05 (1.0 × 10^9^ CFU/kg), LDSB: Mice treated with *Lactobacillus delbrueckii subsp. bulgaricus* (1.0 × 10^9^ CFU/kg). ALT, alanine aminotransferase; AST, aspartate aminotransferase; ALP, alkaline phosphatase; TG, triglycerides; TC, total cholesterol; BUN blood urea nitrogen; ALB, albumin.

**Table 5:** Levels of SOD, NO, CAT, MDA, and GSH-Px in the serum of mouse (*N* = 10).

Group	SOD (U/mL)	NO (µmol/L)	CAT (U/mL)	MDA (µmol/L)	GSH-Px (U/mL)
Normal	128.75 ± 8.12 ^a^	55.36 ± 4.12 ^e^	33.47 ± 2.51 ^a^	6.39 ± 0.46 ^e^	255.37 ± 25.05 ^a^
Model	72.06 ± 3.88 ^d^	133.62 ± 7.23 ^a^	11.86 ± 1.89 ^e^	15.19 ± 0.69 ^a^	134.09 ± 12.38 ^e^
Silymarin	100.32 ± 4.55 ^b^	71.23 ± 5.22 ^d^	26.30 ± 2.24 ^b^	8.02 ± 0.37 ^d^	202.17 ± 15.32 ^b^
LP-HFY05-L	84.21 ± 3.65 ^c^	91.08 ± 6.05 ^c^	18.32 ± 1.93 ^c^	11.03 ± 0.42 ^c^	171.05 ± 13.47 ^c^
LP-HFY05-H	103.14 ± 5.17 ^b^	67.48 ± 5.07 ^d^	27.92 ± 3.03 ^b^	7.78 ± 0.36 ^d^	208.64 ± 16.37 ^b^
LDSB	82.17 ± 3.38 ^c^	108.92 ± 5.40 ^b^	15.32 ± 1.69 ^d^	16.01 ± 0.12 ^b^	145.25 ± 12.28 ^d^

Values are mean ± standard deviation (*N* = 10/group). Sample data in each group come from a normal distribution. The difference in variance between the two groups was significant (*p* < 0.05). ^a–e^ Mean values with different letters over the same column are significantly different (*p* < 0.05) according to Tukey’s honestly significantly different test. Silymarin: Mice treated with silymarin (100 mg/kg), LP-HFY05-L: Mice treated with a low concentration of *Lactobacillus plantarum* HFY05 (1.0 × 10^8^ CFU/kg), LP-HFY05-H: Mice treated with a high concentration of *Lactobacillus plantarum* HFY05 (1.0 × 10^9^ CFU/kg), LDSB: Mice treated with *Lactobacillus delbrueckii subsp. bulgaricus* (1.0 × 10^9^ CFU/kg). SOD, superoxide dismutase; NO, nitric oxide; CAT, catalase; MDA, malondialdehyde; GSH-Px, glutathione peroxidase.

**Table 6:** Serum levels of IL-6, IL-12, TNF-α, and IFN-γ in mice (*N* = 10).

Group	IL-6 (pg/mL)	IL-12 (pg/mL)	TNF-α (pg/mL)	IFN-γ (pg/mL)
Normal	36.77 ± 3.45 ^e^	205.67 ± 16.27 ^e^	22.34 ± 3.06 ^e^	19.87 ± 2.36 ^e^
Model	221.80 ± 18.92 ^a^	805.62 ± 31.20 ^a^	92.54 ± 5.28 ^a^	80.11 ± 4.62 ^a^
Silymarin	75.36 ± 5.30 ^d^	415.28 ± 14.69 ^d^	48.36 ± 3.62 ^d^	32.58 ± 2.87 ^d^
LP-HFY05-L	128.75 ± 11.03 ^c^	581.24 ± 25.33 ^c^	68.20 ± 3.74 ^c^	46.67 ± 3.56 ^c^
LP-HFY05-H	71.39 ± 4.92 ^d^	401.87 ± 18.31 ^d^	44.58 ± 4.02 ^d^	29.87 ± 3.12 ^d^
LDSB	178.45 ± 15.27 ^b^	677.80 ± 22.36 ^b^	80.52 ± 3.68 ^b^	61.02 ± 4.02 ^b^

Values mean ± standard deviation (*N* = 10/group). Sample data in each group come from a normal distribution. The difference in variance between the two groups was significant (*p* < 0.05). ^a–e^ Mean values with different letters over the same column are significantly different (*p* < 0.05) according to Tukey’s honestly significant difference test. Silymarin: Mice treated with silymarin (100 mg/kg), LP-HFY05-L: Mice treated with a low concentration of *Lactobacillus plantarum* HFY05 (1.0 × 10^8^ CFU/kg), LP-HFY05-H: Mice treated with a high concentration of *Lactobacillus plantarum* HFY05 (1.0 × 10^9^ CFU/kg), LDSB: Mice treated with *Lactobacillus delbrueckii subsp. bulgaricus* (1.0 × 10^9^ CFU/kg). IL-6, interluekin-6; TNF-α, tumor necrosis factor-alpha; IFN-γ; interferon-gamma.

**Table 7:** Intestinal fecal microbiota of mice in each group (*N* = 10, 16 rRNA gene copy number, log_10_/g).

Group	Normal	Model	Silymarin	LP-HFY05-L	LP-HFY05-H	LDSB
*Firmicutes*	6.35 ± 0.31 ^e^	8.52 ± 0.47 ^a^	8.12 ± 0.28 ^b^	7.64 ± 0.31 ^c^	7.01 ± 0.21 ^d^	8.05 ± 0.23 ^b^
*Bacteroides*	11.39 ± 0.42 ^a^	7.91 ± 0.35 ^e^	8.26 ± 0.28 ^d^	8.77 ± 0.22 ^c^	9.40 ± 0.42 ^b^	8.23 ± 0.20 ^d^
*Actinobacteria*	1.21 ± 0.15 ^f^	4.32 ± 0.18 ^a^	3.89 ± 0.12 ^b^	2.34 ± 0.11 ^d^	1.81 ± 0.17 ^e^	2.97 ± 0.13 ^c^
*Proteobacteria*	2.36 ± 0.17 ^f^	5.92 ± 0.20 ^a^	4.98 ± 0.18 ^b^	3.69 ± 0.21 ^d^	2.91 ± 0.30 ^e^	4.02 ± 0.19 ^c^
*Bifidobacterium*	2.26 ± 0.11 ^a^	0.35 ± 0.04 ^d^	0.42 ± 0.05 ^d^	0.82 ± 0.08 ^c^	1.89 ± 0.15 ^b^	0.78 ± 0.09 ^c^
*Lactobacillus*	5.23 ± 0.21 ^c^	2.15 ± 0.16 ^e^	2.87 ± 0.19 ^d^	9.41 ± 0.22 ^b^	14.83 ± 0.24 ^a^	4.92 ± 0.24 ^c^
*Enterobacteriaceae*	2.30 ± 0.21 ^e^	6.98 ± 0.31 ^a^	6.34 ± 0.28 ^b^	5.23 ± 0.20 ^d^	2.42 ± 0.25 ^e^	5.51 ± 0.18 ^c^

Values are mean ± standard deviation (*N* = 10/group). Sample data in each group come from a normal distribution. The difference in variance between the two groups was significant (*p* < 0.05). ^a–f^ Mean values with different letters over the same row are significantly different (*p* < 0.05) according to Tukey’s honestly significantly different test. Silymarin: Mice treated with silymarin (100 mg/kg), LP-HFY05-L: Mice treated with a low concentration of *Lactobacillus plantarum* HFY05 (1.0 × 10^8^ CFU/kg), LP-HFY05-H: Mice treated with a high concentration of *Lactobacillus plantarum* HFY05 (1.0 × 10^9^ CFU/kg), LDSB: Mice treated with *Lactobacillus delbrueckii subsp. bulgaricus* (1.0 × 10^9^ CFU/kg).

## References

[1] Chen X., Zhao X., Wang H., Yang Z., Li J., Suo H. (2017). Prevent effects of *Lactobacillus fermentum* HY01 on dextran sulfate sodium-induced colitis in mice. Nutrients.

[2] Wu J., Zhao X.H., Chen J.X., Du M.Y., Kan J.Q. (2013). Isolation and identification of lactic acid bacteria from natural yak yogurt in Tibet plateau pastoral areas of Tibet and western Sichuan. Food Sci..

[3] Liao Y.T., Wu J., Long M., Du M.Y., Kan J.Q. (2015). Screening of dominant lactic acid bacteria from naturally fermented yak milk in Tibetan pastoral areas and optimization of fermentation conditions for yak yogurt production. Food Sci..

[4] Qian Y., Long X.Y., Pan Y.N., Li G.J., Zhao X. (2018). Isolation and identification of lactic acid bacteria (Lactobacillus plantarum YS2) from yak yogurt and its probiotic properties. Biomed. Res..

[5] Qin H., Liang Q., Zhang W.B., Zhang Y., Mi L. (2013). Isolation and identification of lactic acid bacteria with high fermentation performance from naturally fermented yak yogurt in Tibetan pastoral areas of Gansu. Food Sci..

[6] Capozzi V., Fragasso M., Romaniello R., Berbegal C., Russo P., Spano G. (2017). Spontaneous food fermentations and potential risks for human health. Fermentation.

[7] Ripari V., Cecchi T., Berardi E. (2016). Microbiological characterisation and volatiles profile of model, ex-novo, and traditional Italian white wheat sourdoughs. Food Chem..

[8] Mokoena M.P. (2017). Lactic acid bacteria and their bacteriocins: Classification, biosynthesis and applications against uropathogens: A mini-review. Molecules.

[9] Fijan S. (2014). Microorganisms with claimed probiotic properties: An overview of recent literature. Int. J. Environ. Res. Public Health.

[10] Mokoena M.P., Mutanda T., Olaniran A.O. (2016). Perspectives on the probiotic potential of lactic acid bacteria from African traditional fermented foods and beverages. Food Nutr. Res..

[11] Gómez-Zorita S., Aguirre L., Milton-Laskibar I., Fernández-Quintela A., Trepiana J., Kajarabille N., Mosqueda-Solís A., González M., Portillo M.P. (2019). Relationship between changes in microbiota and liver steatosis induced by high-fat feeding—A review of rodent models. Nutrients.

[12] Gupta H., Youn G.S., Shin M.J., Suk K.T. (2019). Role of gut microbiota in hepatocarcinogenesis. Microorganisms.

[13] Fessard A., Kapoor A., Patche J., Assemat S., Hoarau M., Bourdon E., Bahorun T., Remize F. (2017). Lactic fermentation as an efficient tool to enhance the antioxidant activity of tropical fruit juices and teas. Microorganisms.

[14] Cui Y., Hu T., Qu X., Zhang L., Ding Z., Dong A. (2015). Plasmids from food lactic acid bacteria: Diversity, similarity, and new developments. Int. J. Mol. Sci..

[15] Teschke R. (2019). Alcoholic liver disease: Current mechanistic aspects with focus on their clinical relevance. Biomedicines.

[16] Wu D., Cederbaum A.I. (2009). Oxidative stress and alcoholic liver disease. Semin. Liver Dis..

[17] Jeong H.M., Kim D.J. (2019). Bone diseases in patients with chronic liver disease. Int. J. Mol. Sci..

[18] Gómez-Bañuelos E., Mukherjee A., Darrah E., Andrade F. (2019). Rheumatoid arthritis-associated mechanisms of *Porphyromonas gingivalis* and *Aggregatibacter actinomycetemcomitans*. J. Clin. Med..

[19] Drescher H.K., Weiskirchen S., Weiskirchen R. (2019). Current status in testing for nonalcoholic fatty liver disease (NAFLD) and nonalcoholic steatohepatitis (NASH). Cells.

[20] Zhao X., Zhang J., Yi S., Li X., Guo Z., Zhou X., Mu J., Yi R. (2019). *Lactobacillus plantarum* CQPC02 prevents obesity in mice through the PPAR-α signaling pathway. Biomolecules.

[21] Liu L.L., Liu W.S., Han B.Q., Hu J.B. (2010). Protective effects of glucosamine and chitooligosaccharide in alcohol liver injury mice. Period. Ocean Univ. China.

[22] Sun J., Wang F., Li H., Zhang H., Jin J., Chen W., Pang M., Yu J., He Y., Liu J. (2015). Neuroprotective effect of sodium butyrate against cerebral ischemia/reperfusion injury in mice. Biomed. Res. Int..

[23] Li C., Tan F., Yang J., Yang Y., Gou Y., Li S., Zhao X. (2019). Antioxidant effects of *Apocynum venetum* tea extracts on d-galactose-induced aging model in mice. Antioxidants.

[24] Liu B., Zhang J., Sun P., Yi R., Han X., Zhao X. (2019). Raw Bowl tea (Tuocha) polyphenol prevention of nonalcoholic fatty liver disease by regulating intestinal function in mice. Biomolecules.

[25] Gao B., Bataller R. (2011). Alcoholic liver disease: Pathogenesis and new therapeutic targets. Gastroenterology.

[26] Colica C., Boccuto L., Abenavoli L. (2017). Silymarin: An option to treat non-alcoholic fatty liver disease. World J. Gastroenterol..

[27] Wang M., Niu J., Ou L., Deng B., Wang Y., Li S. (2019). Zerumbone protects against carbon tetrachloride (CCl_4_)-induced acute liver injury in mice via inhibiting oxidative stress and the inflammatory response: Involving the TLR4/NF-κB/COX-2 pathway. Molecules.

[28] Iweala E.E.J., Evbakhavbokun W.O., Maduagwu E.N. (2019). Antioxidant and hepatoprotective effect of *Cajanus cajan* in N-nitrosodiethylamine-induced liver damage. Sci. Pharm..

[29] Albasher G., Almeer R., Al-Otibi F.O., Al-Kubaisi N., Mahmoud A.M. (2019). Ameliorative effect of *Beta vulgaris* root extract on chlorpyrifos-induced oxidative stress, inflammation and liver injury in rats. Biomolecules.

[30] Wang R., Yang Z., Zhang J., Mu J., Zhou X., Zhao X. (2019). Liver injury induced by carbon tetrachloride in mice is prevented by the antioxidant capacity of Anji White tea polyphenols. Antioxidants.

[31] Tang B.B., Hu D.H. (2016). Effect of early bedside hemofiltration on systemic inflammatory state as well as liver and kidney function in patients with severe acute pancreatitis. J. Hainan Med. Univ..

[32] Farbiszewski R., Radecka A., Chwiecko M., Holownia A. (1992). The effect of heparegen on antioxidant enzyme activities in ethanol-induced liver injury in rats. Alcohol.

[33] Huang Q.H., Xu L.Q., Liu Y.H., Wu J.Z., Wu X., Lai X.P., Li Y.C., Su Z.R., Chen J.N., Xie Y.L. (2017). Polydatin protects rat liver against ethanol-induced injury: Involvement of CYP2E1/ROS/Nrf2 and TLR4/NF-κB p65 pathway. Evid. Based Complement. Alternat. Med..

[34] Li Y.G., Ji D.F., Chen S., Hu G.Y. (2008). Protective effects of sericin protein on alcohol-mediated liver damage in mice. Alcohol Alcohol..

[35] Wang X., Liu M., Zhang C., Li S., Yang Q., Zhang J., Gong Z., Han J., Jia L. (2018). Antioxidant activity and protective effects of enzyme-extracted *Oudemansiella radiata* polysaccharides on alcohol-induced liver injury. Molecules.

[36] Sun X., Wang P., Yao L.P., Wang W., Gao Y.M., Zhang J., Fu Y.J. (2018). Paeonol alleviated acute alcohol-induced liver injury via SIRT1/Nrf2/NF-κB signaling pathway. Environ. Toxicol. Pharmacol..

[37] Clemens M.G. (1999). Nitric oxide in liver injury. Hepatology.

[38] Arteel G.E. (2003). Oxidants and antioxidants in alcohol-induced liver disease. Gastroenterology.

[39] An L., Wang X., Cederbaum A.I. (2012). Cytokines in alcoholic liver disease. Arch. Toxicol..

[40] Eipel C., Hardenberg J., Negendank S., Abshagen K., Vollmar B. (2009). Thrombopoietin limits IL-6 release but fails to attenuate liver injury in two hepatic stress models. Eur. J. Gastroenterol. Hepatol..

[41] Cheng L., Wang J., Li X., Xing Q., Du P., Su L., Wang S. (2011). Interleukin-6 induces Gr-1+CD11b+ myeloid cells to suppress CD8+ T cell-mediated liver injury in mice. PLoS ONE.

[42] Cheng L., Du X., Wang Z., Ju J., Jia M., Huang Q., Xing Q., Xu M., Tan Y., Liu M. (2014). Hyper-IL-15 suppresses metastatic and autochthonous liver cancer by promoting tumour-specific CD8+ T cell responses. J. Hepatol..

[43] Zhou H.C., Wang H., Shi K., Li J.M., Zong Y., Du R. (2019). Hepatoprotective effect of baicalein against acetaminophen-induced acute liver injury in mice. Molecules.

[44] Liu B., Fang Y., Yi R., Zhao X. (2019). Preventive effect of blueberry extract on liver injury induced by carbon tetrachloride in mice. Foods.

[45] Qiao J.Y., Li H.W., Liu F.G., Li Y.C., Tian S., Cao L.H., Hu K., Wu X.X., Miao M.S. (2019). Effects of *Portulaca Oleracea* Extract on Acute Alcoholic Liver Injury of Rats. Molecules.

[46] Alexaki V.I., Charalampopoulos I., Kampa M., Vassalou H., Theodoropoulos P., Stathopoulos E.N., Hatzoglou A., Gravanis A., Castanas E. (2004). Estrogen exerts neuroprotective effects via membrane estrogen receptors and rapid Akt/NOS activation. FASEB J..

[47] Wickman A., Jonsdottir I.H., Bergstrom G., Hedin L. (2002). GH and IGF-I regulate the expression of endothelial nitric oxide synthase (eNOS) in cardiovascular tissues of hypophysectomized female rats. Eur. J. Endocrinol..

[48] Hayashi Y., Abe M., Murai A., Shimizu N., Okamoto I., Katsuragi T., Tanaka K. (2005). Comparison of effects of nitric oxide synthase (NOS) inhibitors on plasma nitrite/nitrate levels and tissue NOS activity in septic organs. Microbiol. Immunol..

[49] Lin H.I., Wang D., Leu F.J., Chen C.F., Chen H.I. (2004). Ischemia and reperfusion of liver induces eNOS and iNOS expression: Effects of a NO donor and NOS inhibitor. Chin. J. Physiol..

[50] Liu B., Li J., Yi R., Mu J., Zhou X., Zhao X. (2019). Preventive effect of alkaloids from *Lotus plumule* on acute liver injury in mice. Foods.

[51] Kanai S., Okano H. (1998). Mechanism of the protective effects of sumac gall extract and gallic acid on the progression of CCl_4_-induced acute liver injury in rats. Am. J. Chin. Med..

[52] Wheeler M.D., Nakagami M., Bradford B.U., Uesugi T., Mason R.P., Connor H.D., Dikalova A., Kadiiska M., Thurman R.G. (2001). Overexpression of manganese superoxide dismutase prevents alcohol-induced liver injury in the rat. J. Biol. Chem..

[53] Kobaek-Larsen M., Baatrup G., Notabi M.K., El-Houri R.B., Pipó-Ollé E., Christensen Arnspang E., Christensen L.P. (2019). Dietary polyacetylenic oxylipins falcarinol and falcarindiol prevent inflammation and colorectal neoplastic transformation: A mechanistic and dose-response study in a rat model. Nutrients.

[54] Rusciano M.R., Sommariva E., Douin-Echinard V., Ciccarelli M., Poggio P., Maione A.S. (2019). CaMKII activity in the inflammatory response of cardiac diseases. Int. J. Mol. Sci..

[55] Wu Y.F., Zhang X.L., Zhang Y.X., Song Q.M., Hua X.M., Jia D.M., Wang Y.H. (2015). Ameliorating alcoholic liver injury by adjusting intestinal microbiota with probiotics. Microbiology.

[56] Miao Y.L., Xiao Y.L., Duan L.P., Li X.Y., Chen L.F., Li H.N. (2009). The study of oligomeric nucleotide chip to detect gene expression profile of ulcerative colitis patients. Chin. J. Digestol..

[57] Neuman M.G., Brenner D.A., Reherman N.B., Taieb J., Chollet-Martin S., Cohard M., Garaud J.J., Poynard T., Katz G.G., Cameron R.G. (2001). Mechanisms of alcoholic liver disease: Cytokines. Alcohol Clin. Exp. Res..

[58] Inokuchi S., Tsukamoto H., Park E., Liu Z.X., Brenner D.A., Seki E. (2011). Toll-like receptor 4 mediates alcohol-induced steato hepatitis through bone marrow-derived and endogenous liver cells in mice. Alcohol Clin. Exp. Res..

[59] Bajaj J.S., Hylemon P.B., Ridlon J.M., Heuman D.M., Daita K., White M.B., Monteith P., Noble N.A., Sikaroodi M., Gillevet P.M. (2012). Colonic mucosal microbiome differs from stool microbiome in cirrhosis and hepatic encephalopathy and is linked to cognition and inflammation. Am. J. Physiol. Gastrointest. Liver Physiol..

[60] Bull-Otterson L., Feng W., Kirpich I., Wang Y., Qin X., Liu Y., Gobejishvili L., Joshi-Barve S., Ayvaz T., Petrosino J. (2013). Metagenomic analyses of alcohol induced pathogenic alterations in the intestinal microbiome and the effect of Lactobacillus rhamnosus GG treatment. PLoS ONE.

[61] Ripari V. (2019). Techno-functional role of exopolysaccharides in cereal-based, yogurt-like beverages. Beverages.

